# A brain DNA co‐methylation network analysis of psychosis in Alzheimer's disease

**DOI:** 10.1002/alz.14501

**Published:** 2025-02-12

**Authors:** Morteza Kouhsar, Luke Weymouth, Adam R. Smith, Jennifer Imm, Claudia Bredemeyer, Yehani Wedatilake, Ali Torkamani, Sverre Bergh, Geir Selbæk, Jonathan Mill, Clive Ballard, Robert A. Sweet, Julia Kofler, Byron Creese, Ehsan Pishva, Katie Lunnon

**Affiliations:** ^1^ Department of Clinical and Biomedical Sciences Faculty of Health and Life Sciences University of Exeter Exeter Devon UK; ^2^ Norwegian National Centre for Aging and Health Vestfold Hospital Trust Tønsberg Norway; ^3^ Research Centre for Age‐related Functional Decline and Disease Innlandet Hospital Trust Ottestad Norway; ^4^ The Scripps Research Institute La Jolla California USA; ^5^ Department of Geriatric Medicine Oslo University Hospital Nydalen Oslo Norway; ^6^ Department of Psychiatry University of Pittsburgh Pittsburgh Pennsylvania USA; ^7^ Department of Pathology University of Pittsburgh Pittsburgh Pennsylvania USA; ^8^ Division of Psychology Department of Life Sciences Brunel University London Uxbridge UK; ^9^ Department of Psychiatry and Neuropsychology School for Mental Health and Neuroscience (MHeNs) Faculty of Health Medicine and Life Sciences (FHML) Maastricht University Maastricht The Netherlands

**Keywords:** Alzheimer's disease (AD), brain, DNA methylation, epigenetics, methylation quantitative trait loci (mQTLs), pathways, psychosis, schizophrenia, weighted gene correlation network analysis (WGCNA)

## Abstract

**INTRODUCTION:**

The presence of psychosis in Alzheimer's disease (AD) is suggested to be associated with distinct molecular and neuropathological profiles in the brain.

**METHODS:**

We assessed brain DNA methylation in AD donors with psychosis (AD+P) and without psychosis (AD−P) using the EPIC array. Weighted gene correlation network analysis identified modules of co‐methylated genes in a discovery cohort (PITT‐ADRC: *N* = 113 AD+P, *N* = 40 AD−P), with validation in an independent cohort (BDR: *N* = 79 AD+P, *N* = 117 AD−P), with Gene Ontology and cell‐type enrichment analysis. Genetic data were integrated to identify methylation quantitative trait loci (mQTLs), which were co‐localized with GWAS for related traits.

**RESULTS:**

We replicated one AD+P associated module, which was enriched for synaptic pathways and in excitatory and inhibitory neurons. mQTLs in this module co‐localized with variants associated with schizophrenia and educational attainment.

**DISCUSSION:**

This represents the largest epigenetic study of AD+P to date, identifying pleiotropic relationships between AD+P and related traits.

**Highlights:**

- DNA methylation was assessed in the prefrontal cortex in subjects with AD+P and AD−P.
- WGCNA identified six modules of co‐methylated loci associated with AD+P in a discovery cohort.
- One of the modules was replicated in an independent cohort.
- This module was enriched for synaptic genes and in excitatory and inhibitory neurons.
- mQTLs mapping to genes in the module co‐localized with GWAS loci for schizophrenia and educational attainment.

## 1 BACKGROUND

Psychosis (broadly speaking delusions and hallucinations) has a cumulative disease prevalence of around 40% in Alzheimer's disease (AD). Psychosis in AD (AD+P) is associated with faster cognitive and functional decline, mortality, hospitalization, a shorter time to institutionalization, and increased caregiver distress.^1^, ^2^, ^3^, ^4^, ^5^, ^6^, ^7^, ^8^

Despite the clear benefits that effective management of AD+P would bring, there is currently a critical treatment gap. Atypical antipsychotics are frequently used but have limited benefits and are associated with a significantly increased risk of stroke, pneumonia, falls, and mortality.^9^ In terms of drug evaluation, most trials are of existing antipsychotics used to treat schizophrenia,^10^, ^11^, ^12^ and the pipeline of emerging therapies is very sparse. The 5HT2A inverse agonist pimavanserin has been licensed in the United States for the treatment of Parkinson's disease psychosis,^13^ and there is some evidence of benefit for AD+P.^14^ It is, however, imperative that we better understand specific disease mechanisms underlying AD+P, so that novel and more targeted treatments can be developed.

Neuropathological associations with AD+P include increased neurofibrillary tangles (NFTs) and hyperphosphorylated tau burden in the brain (particularly in the prefrontal cortex), the presence of co‐morbid TAR DNA‐binding protein 43 (TDP‐43) pathology and higher total tau in cerebrospinal fluid (CSF).^15^, ^16^, ^17^, ^18^, ^19^, ^20^ However, it has been reported that neuropathology explains only ∼18% of the variance in AD+P,^20^ and a growing body of evidence supports a genetic basis for AD+P. Early studies in familial disease estimated the heritability of AD+P at 61%, and more recent studies have capitalized on advances in technology to examine genome‐wide significant linkage, copy number variants, and polygenic risk scores (PRS).^21^, ^22^, ^23^, ^24^, ^25^, ^26^ A 2021 genome‐wide association study (GWAS) estimated SNP heritability at 18% to 31%, which roughly equates to that of schizophrenia and major depressive disorder.^27^ Together these estimates suggest that the study of other factors besides common genetic variation and neuropathology may help elucidate the mechanisms underlying AD+P.

The reversible regulation of various genomic functions can occur independently of DNA sequence variation via epigenetic mechanisms. The best characterized epigenetic process regulating gene expression is DNA methylation. Several epigenome‐wide association studies (EWAS) exploring DNA methylation patterns have been undertaken in AD brain^28^, ^29^, ^30^, ^31^, ^32^, ^33^ and blood,^34^, ^35^, ^36^, ^37^ with several robust loci identified from recent meta‐analyses.^33^, ^38^ In a recent study, we explored DNA methylomic variation in AD+P *post mortem* brain samples by leveraging existing data generated in individuals with *ante mortem* neuropsychiatric assessments, identifying three significant differentially methylated regions (DMRs) associated with AD+P (*TBX15*, *WT1*, and *FAM53*). There was also evidence of hypomethylation at the *AS3MT* locus.^39^ Interestingly, altered DNA methylation is robustly observed in *AS3MT* in schizophrenia, and this molecular‐level evidence reflects broader observations in the genetic literature of links between AD+P and neuropsychiatric disorders that occur earlier in life.^27^, ^40^, ^41^

**FIGURE 1 alz14501-fig-0001:**
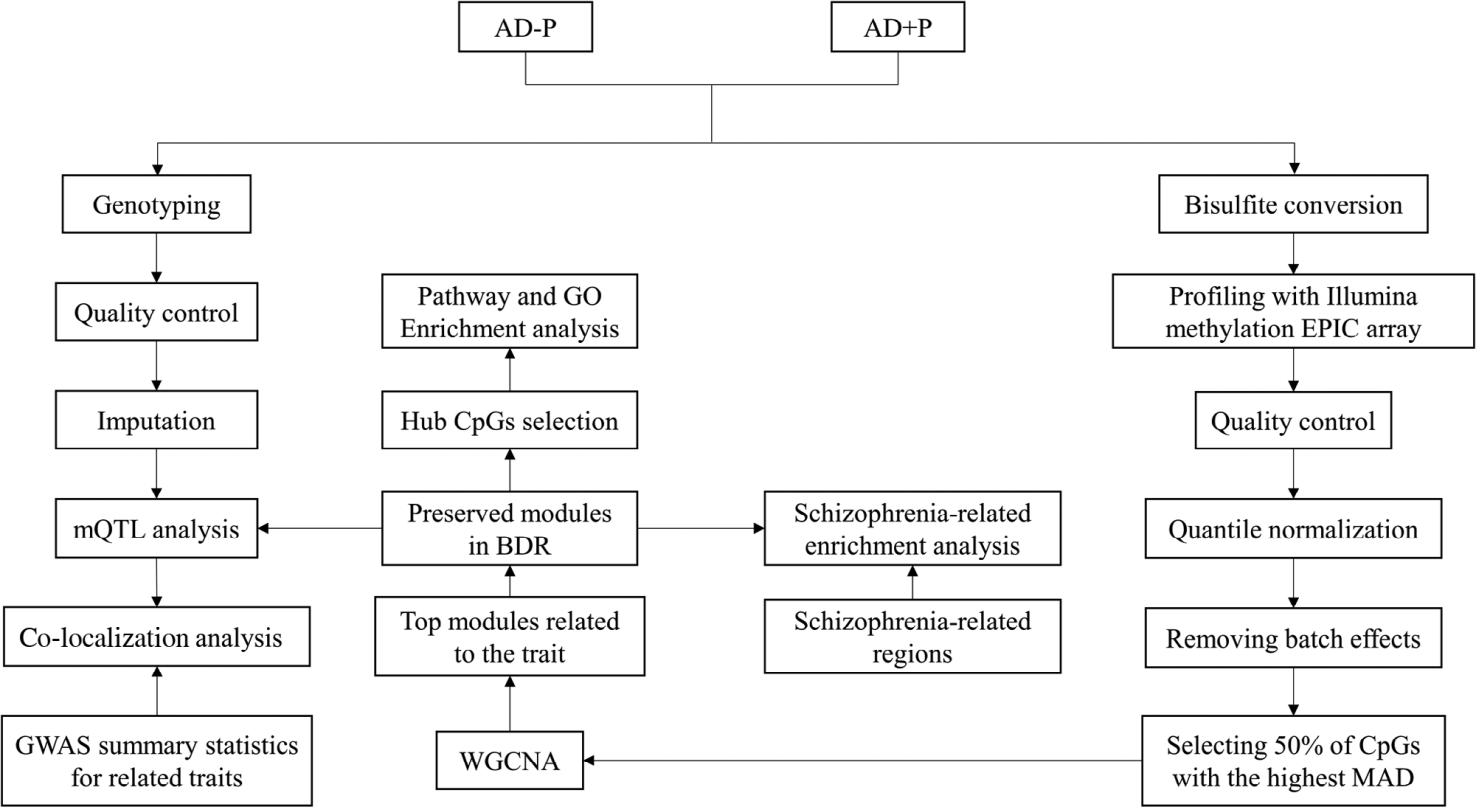
Overall workflow of study. Abbreviations: AD+P, Alzheimer's disease with psychosis; AD−P, Alzheimer's disease without psychosis; GO, Gene Ontology; GWAS, genome‐wide association study; MAD, median absolute deviation; mQTLs, methylation quantitative trait loci; WGCNA, weighted gene correlation network analysis.

Taken together, previous epigenomic, genomic, and neuropathological studies have provided a growing body of evidence that AD+P has a distinct neurobiological profile. Here, we have profiled genome‐wide DNA methylation in the dorsolateral prefrontal cortex (DLPFC) in a discovery cohort of 153 individuals with *ante mortem* neuropsychiatric evaluations, performing weighted gene correlation network analysis (WGCNA) to identify modules of co‐methylated loci associated with the presence of psychosis, with validation in an independent dataset of 196 individuals. Subsequent Gene Ontology (GO) and cell‐type enrichment analysis were then performed on the replicated module. To identify pleiotropic relationships between AD+P and other related traits, we identified methylation quantitative trait loci (mQTLs) corresponding to CpG sites in the replicated AD+P associated module, co‐localizing these with genomic regions reported in the GWAS for these other traits (see Figure 1 for an overview of the study). Together, this represents the largest and most comprehensive study to date of DNA methylation in the context of AD+P.

## 2 METHODS

### 2.1 Study cohorts

Our study utilized *post mortem* brain samples with corresponding *ante mortem* neuropsychiatric assessments from the University of Pittsburgh Alzheimer's Disease Research Centre (PITT‐ADRC) and Brains for Dementia Research (BDR) cohorts.

PITT‐ADRC is a long‐standing longitudinal research cohort of normal controls and preclinical and clinical dementia subjects with neuroimaging, genetic, and neuropathological assessments.^42^ AD pathology is assessed and classified using the Consortium to Establish a Registry for Alzheimer's Disease (CERAD) neurotic plaque density score, Braak NFT stages, and National Institute on Aging and Reagan Institute (NIA‐RI) criteria. Cases obtained since 2012 are also routinely assigned a Thal phase and ABC score based on the revised 2012 NIA‐Alzheimer's Association (AA) criteria. All cases were also screened for the presence of concurrent TDP‐43 and alpha‐synuclein pathologies.

**RESEARCH IN CONTEXT**

1. **Systematic review**: There is mounting evidence that AD+P results in a neurobiological profile that is distinct from AD−P, with evidence of altered genomic regulation at genetic, epigenetic, and transcriptomic levels, in addition to synaptic protein and neuropathological differences.
2. **Interpretation**: We used WGCNA to identify modules of co‐methylated loci associated with AD+P in the prefrontal cortex, with one of these modules replicated in an independent dataset. This module was particularly enriched for genes relating to the synapse, and cell type enrichment analysis showed a significant enrichment in excitatory neurons. mQTLs for genes within this module co‐localized with SNPs associated with schizophrenia and educational attainment.
3. **Future directions**: Our study combined genetic and epigenetic data to identify pleiotropic relationships between AD+P and the traits of schizophrenia and educational attainment. Since this study used bulk tissue, future studies should explore this relationship in distinct cell types in the brain.

BDR, established in 2008, is a unique resource for dementia research within the UK.^43^ It contains *post mortem* brain samples with detailed clinical (including neuropsychiatric) assessments, taken annually during life using a standardized protocol.^44^, ^45^ It comprises six national brain banks using identical standard operating procedures to collect and store brain material rapidly after death, ensuring high‐quality material for molecular studies.

### 2.2 Dementia and psychosis case ascertainment and cohort harmonization

Both BDR and PITT‐ADRC samples were categorized based on their cognitive status. Specifically, samples exhibiting a Mini‐Mental State Examination (MMSE) score of less than 24 or a last Clinical Dementia Rating (CDR) score ≥ 1 were classified as having dementia. Within this dementia group, samples meeting both the criteria of CERAD score ≥ 2 and Braak NFT stage ≥ III were categorized as AD. In instances where CERAD information was unavailable, samples with a Braak NFT stage ≥ V were also included in the AD group.[Fig alz14501-fig-0001]

Psychotic symptoms were rated using the CERAD Behavioral Rating Scale (BRS)^46^ in the PITT‐ADRC cohort and the Neuropsychiatric Inventory (NPI)^47^ in the BDR cohort. Because of the different rating scales used, psychosis status was coded as present or absent based on the frequency of reported symptoms over the past month prior to the assessment (derived from ratings on each of the respective measures). To be classified as AD+P, symptoms had to be recurrent over the past month as this phenotype has shown the highest heritability.^21^ The frequency coding for the BRS and NPI are slightly different, but both use the past month prior to assessment as the reference period. A comparable frequency rating for recurrent symptoms is “at least 3 times [in the past month]” for the BRS and “at least once a week [in the past month]” for the NPI (note the severity rating of the NPI was not considered so that scores better matched the BRS, which rates frequency only). Therefore, PITT‐ADRC cases with delusions or hallucinations rated as present at least three times in the month prior to assessment were classified as AD+P, while BDR cases with delusions or hallucinations at least once a week for the month prior to assessment were classified as AD+P. In both cohorts, AD without psychosis (AD−P) status required scores of 0 (ie, no symptoms) at all available assessment time points.

We opted to use the PITT‐ADRC cohort for discovery and the BDR for replication, for a number of reasons. First, the PITT‐ADRC cohort has been used extensively to explore psychosis in AD in *post mortem* tissue, since it has been used for genomic studies,^48^ pathological studies,^15^ and proteomic studies.^49^ Second, the samples all come from a single brain bank, while BDR samples are drawn from a network of six brain banks in the UK. Third, the *post mortem* interval was significantly lower in the PITT‐ADRC samples (mean = 6.75 h) than in the BDR samples we used. Fourth, the number of *ante mortem* neuropsychiatric assessments was higher in PITT‐ADRC than BDR. Finally, we specifically generated DNA methylomic data in PITT‐ADRC for the specific purpose of our study, meaning that we organized our experimental workflow to minimize batch effects and ensure that AD+P and AD−P samples were randomly processed across arrays, which were run as a single batch.

### 2.3 Sample preparation

For the PITT‐ADRC cohort, DLPFC tissue was milled to a fine powder consistency using a pre‐cooled pestle while the sample tube sat in a basin containing liquid nitrogen. Subsequently, genomic DNA was isolated from 30 mg of DLPFC tissue using the Qiagen AllPrep DNA/RNA/miRNA Universal Kit.

### 2.4 Bisulfite treatment and Illumina Infinium BeadArray

For the PITT‐ADRC cohort, genomic DNA was bisulfite converted using the Zymo EZ‐96 DNA Methylation‐Gold Kit. The bisulfite‐converted DNA samples were analyzed on the Illumina Infinium Human Methylation EPIC BeadChip Array (EPIC array), which generates a quantitative measurement of DNA methylation for > 850,000 CpG sites. EPIC array data had already been generated on the BDR cohort,^38^ so for our analyses we used the raw data for the subset of this BDR cohort that met the criteria outlined in Section 2.2.[Table alz14501-tbl-0001]

We conducted comprehensive data pre‐processing and normalization on both the PITT‐ADRC and BDR cohorts to ensure the harmonization of data. We imported the raw intensity IDAT files into R (version 3.5.2) using the readEPIC function from the wateRmelon R package (version 1.26.0).^50^ Subsequently, both MethylumiSet and RGChannelSet objects were generated using the wateRmelon and minfi (version 1.28.4)^51^ R packages, respectively. For data quality control (QC) we removed samples from the analysis if the median signal intensity < 750, the bisulfite conversion efficiency < 80% (using the “bscon” function from wateRmelon), or where there was a mismatch between predicted and reported sex (using the “getSex” function in minfi). The “pfilter” function in wateRmelon was used to remove samples with detected *p* values < 0.05, and probes with a bead count <3 in over 5% of samples. Outlier samples were identified and removed using the “outlyx” function within wateRmelon. Subsequently, quantile normalization was applied using the “dasen” function in the wateRmelon package. Following data pre‐processing and quality control, we had 153 samples in the PITT‐ADRC cohort that were taken forward for analysis (113 AD+P, 40 AD−P) and 196 samples in the BDR cohort for validation (79 AD+P, 117 AD−P) (Table 1).

**TABLE 1 alz14501-tbl-0001:** Demographics for samples used in study. Shown for the discovery (PITT‐ADRC) and independent (BDR) cohort are the number of samples, mean age (standard deviation), number of females, number of males, and mean Braak stage (standard deviation) for AD donors with psychosis (AD+P) and without psychosis (AD−P).

Cohort	Group	Age (SD)	*N* female	*N* male	Braak stage (SD)
PITT‐ADRC	AD−P	77.94 (10.76)	17	23	5.45 (0.93)
	AD+P	80.79 (9.55)	62	51	5.71 (0.61)
BDR	AD−P	83.31 (7.71)	64	53	5.18 (0.94)
	AD+P	82.23 (8.24)	29	50	5.45 (0.78)

### 2.5 Illumina Global Screening Array and imputation

Genotyping was conducted using the Illumina Global Screening Array (GSA), which covers >500,000 common SNPs. The raw IDAT files were imported into the Illumina Genome Studio 2.0 software^52^ and subsequently converted into the PLINK binary format. We employed PLINK 1.9^53^ to filter out any samples with >5% missing values and SNPs with > 1% missing values. Moreover, SNPs with a minor allele frequency (MAF) < 0.05 and Hardy–Weinburg equilibrium *p* value < 1. × 10^−5^ were excluded from our analysis. Samples were also checked to ensure there was no discrepancy between their predicted and reported sexes. Data were merged with the 1000 Genome Project data, and principal component analysis (PCA) was implemented to determine sample ethnicity, with non‐European samples filtered out of subsequent analyses. The relatedness among the samples was calculated by the kinship coefficient using KING (version 2.2.7).^54^ In instances where the kinship coefficient exceeded 0.25 for any pair of samples, one sample from each pair was systematically removed.

The data were uploaded to Michigan Imputation Server and imputed using Minimac4^55^ and 1000 Genomes reference panel (phase 3, version 5). The imputed data, separated by chromosomes, were then consolidated into a unified VCF file using BCFtools (version 1.9)^56^. Triallelic SNPs were diligently removed from this dataset using VCFtools (version 0.1.16).^57^ Finally, the VCF file was converted into the PLINK binary format to facilitate further in‐depth analysis. Following imputation and QC we retained 6,568,087 SNPs in 151 samples (111 AD+P, 40 AD−P) in the PITT‐ADRC cohort.

### 2.6 WGCNA analysis

Our primary analysis focused on the execution of WGCNA on the PITT‐ADRC cohort with subsequent validation of its findings in the BDR cohort. Using the normalized beta value matrix from the methylation data obtained from the PITT‐ADRC, the effects of potentially confounding variables (age, sex, Braak NFT stage, estimated cell type proportion [calculated using the minfi package] and plate) were regressed out for each CpG site using a linear regression model. Age, sex, and cell proportions are all known to have a significant impact on DNA methylation levels, and we routinely control for these in our DNA methylomic studies in AD brain (eg, Lunnon et al., Smith et al., Shireby et al.^28^, ^33^, ^38^). As AD+P donors are reported to have increased NFT and hyperphosphorylated tau burden in the brain compared to AD−P,^17^, ^18^ we included Braak NFT stage as a co‐variate to ensure we were removing any potential confounding arising from the AD+P group potentially having more tau pathology. Hierarchical clustering analysis was performed using both Euclidean distance and correlation distance (as similarity metrics between samples) with no consistent outlier samples identified (Figure S1). Next, we computed the median absolute deviation (MAD) for each CpG site, and subsequently the top 50% of CpG sites with the highest MAD scores were retained to construct the co‐methylation network (*N* = 395,274 probes). The BDR data were processed in an identical manner for later validation purposes (Section 2.7).

Then we used a block‐wise approach within the WGCNA package^58^ to both construct the co‐methylation network and identify modules. An unsigned network was constructed employing a soft threshold power of three and a maximum block size of 30,000 (Figure S2). Similar to our previous studies,^35^, ^37^, ^59^ an unsigned network was used because it allows for the detection of clusters of both hyper‐ and hypomethylated CpG sites that exhibit coordinated methylation changes that together regulate gene expression. Subsequently, all modules with a minimum size of 100 probes were extracted using the default approach integrated into WGCNA. Next, the “moduleEigengenes” function was employed to establish the module eigengene (ME), defined as the first principal component (PC) within each identified module. As we were investigating a single indicator variable, we employed a *t*‐test to assess the module–trait relationship, distinguishing between the AD+P and AD−P groups for each ME vector. Additionally, we computed module membership (MM) for every CpG site within each module by calculating the Pearson correlation between its methylation values and the corresponding ME. Probe significance (PS) was defined as the Pearson correlation between our trait (ie, the presence of psychosis) and the methylation values of each CpG site within each module. Subsequently, we evaluated the Pearson correlation between MM and PS across all CpG sites within each module. Modules that showed a significant MM–PS correlation and a module–trait (eg, AD+P) relationship (*p* < 0.05) were selected as the AD+P‐related modules within the PITT‐ADRC dataset and taken forward for validation. Hub genes were defined as the top 25% of CpG sites, with the highest MM and PS in a module, representing the most integral probes within that module.

### 2.7 Module preservation analysis

The module preservation function in the WGCNA package was used to assess the preservation of the co‐methylation modules identified in the PITT‐ADRC cohort in the BDR cohort. Preservation levels were quantified using the Z‐summary statistic. Modules scoring a Z‐summary statistic > 10 were classified as highly preserved, with those scoring between 5 and 10 being categorized as moderately preserved. Modules with a Z‐summary statistic < 5 were deemed not to be preserved between the two datasets. The analysis utilized 1000 permutations, with all remaining parameters set to default. We considered modules to be replicated in the BDR cohort if they were (1) highly preserved (Z‐summary > 10), (2) had a significant MM–PS correlation, and (3) had a significant module–trait correlation (*p* < 0.05). These replicated modules were taken forward for downstream analysis.

### 2.8 Gene ontology and cell type enrichment analyses

The “gometh” function in the missMethyl package (version 1.30.0)^60^ was used for GO and Kyoto Encyclopedia of Genes and Genomes (KEGG) pathway enrichment analysis for any preserved and replicated module associated with AD+P in both cohorts. This function uses the annotated gene symbols corresponding to the CpG sites in a module of interest in the Illumina EPIC manifest data. The prior probability was set to true to adjust the results based on the number of probes per gene. We also used gene symbols corresponding to all CpG sites (*N* = 395,274) as a background. Protein–protein interaction (PPI) networks^61^, ^62^ were generated for genes < 1 kb from CpG sites located in the modules of interest. To determine which cell types might be driving the DNA methylation changes in the modules, we employed expression‐weighted cell‐type enrichment analysis (EWCE) using functions within the EWCE R package (version 1.6.0).^63^ Single‐cell RNA sequencing data generated in the DLPFC in the Religious Orders Study and Memory and Aging Project (ROSMAP)^64^ was loaded into R using the “Read10x()” function in the Seurat package (version 4.3.0.1).^65^ Prior to cell‐type enrichment analysis, gene symbols that did not have a Hugo Gene Nomenclature Committee (HGNC) approved symbol were fixed using the “fix_bad_hgnc_symbols()” function. Uninformative genes (ie, genes that do not have 1:1 human orthologs, low‐variance genes, and non‐differentially expressed genes across cells) were also removed using the “drop_uninformative_genes()” function in EWCE. Finally, cell‐type enrichment analysis was performed assessing the expression levels of genes annotated to the CpG sites in a module of interest in the single‐cell RNA sequencing reference data, applying the “bootstrap_enrichment_test()” function. This was performed for all CpG sites in a module of interest, and again for hub CpG sites in a module of interest.

### 2.9 mQTL identification and co‐localization analysis

The genotyping and the regressed‐out DNA methylation data we generated in the PITT‐ADRC cohort were used to identify mQTLs using the MatrixEQTL package (version 2.3).^66^ An additive linear model was used to capture the influence of allele count on DNA methylation, including the first three PCs extracted from the genotype data as covariates to account for ethnicity‐related effects in the model. For all CpG probes within preserved AD+P‐related modules, we identified mQTLs both in *cis* (within ± 1 Mb from each CpG location) and in *trans* (> 1 Mb from each CpG location but still within the same chromosome) for SNP–probe pairs, applying a pre‐defined *p* value threshold of 1.0 × 10^−5^.

We then tested for potential pleiotropic effects between the mQTLs in the preserved AD+P‐associated modules and genetic variants reported for other related traits. To this end we gathered and examined comprehensive summary statistics from several GWASs covering a wide range of related traits in European populations. These studies included AD,^67^ schizophrenia, panic disorder,^69^ anxiety,^70^ educational attainment,^71^ bipolar disorder,^72^ major depressive disorder, Lewy body dementia,^74^ AD+P,^48^ and reaction time^75^ (Table S1). In instances where specific studies provided lead SNPs instead of genomic regions, we utilized the PLINK software to calculate pairwise linkage disequilibrium (LD) between each lead SNP and all SNPs located within a 250‐kb radius. To delineate the region around each lead SNP, we considered the furthest SNP with an *r* ^2^ value greater than 0.6 within a maximum distance of 250 kb.

Co‐localization analysis was conducted on both the *cis* and *trans* mQTLs associated with each CpG in the preserved AD+P‐related modules and GWAS regions for related traits using the coloc package in R (version 5.1.0.1).^76^ We utilized default prior probability settings and employed the “Chromosome:Position” identifier to identify common SNPs shared between mQTLs and summary statistics. The coloc package calculates multiple posterior probabilities aimed at quantifying the degree of support for five distinct hypotheses: H_0_ (there are no causal variants for either trait), H_1_ (there is a causal SNP for the related trait in the tested study), H_2_ (there is a causal SNP associated with DNA methylation), H_3_ (there are two distinct causal variants, one for DNA methylation and one for the related trait in the tested study), H_4_ (there is a single causal variant common to DNA methylation and the related trait in the tested study). We used a posterior probability for H_3_ + H_4_ > 0.9 as a cut‐off for deeming that the regions were co‐localized.

To minimize the chances that any identified co‐localized regions were false positives, we generated a co‐localization *p* value. In this process, for each identified region where the combined posterior probability (H3 + H4) exceeded 0.9, we randomly generated 1000 regions of identical length to the identified region, using the same LD thresholds as the identified region's lead SNPs. Subsequently, we performed a co‐localization analysis on all 1000 randomly generated regions and then established a co‐localization *p* value using the following formula:

$$\begin{array}{ccc} & & \mathrm{Co}\;-\;\mathrm{localization}\;p\;\mathrm{value}\\ & & \;=\;\frac{\mathrm{Number}\;\mathrm{of}\;\mathrm{random}\;\mathrm{regions}\;\mathrm{with}\;H3+H4>0.9}{\mathrm{Total}\;\mathrm{number}\;\mathrm{of}\;\mathrm{random}\;\mathrm{regions}} \end{array}$$

We then deemed the co‐localization result for the identified region a true result if the calculated *p* value was < 0.05 (indicating that at least 95% of randomly generated regions had a combined posterior probability that did not meet our co‐localization criteria).

### 2.10 Schizophrenia GWAS and EWAS enrichment analysis

We performed enrichment analysis for CpG sites nominated in schizophrenia EWAS (based on 2104 and 50 CpG sites from previous studies, respectively^77^, ^78^) by comparing them with CpG sites in a module of interest using a Fisher exact test. Since our data are derived from the Illumina EPIC array, while the two schizophrenia studies are based on the 450K array, we used the CpG sites shared between these two arrays as the background. A contingency matrix was created using the list of CpG sites in a module of interest, schizophrenia‐related CpG sites (nominated from the two EWAS), and the background CpG sites. A Fisher’ exact test was then performed to determine whether a module of interest was enriched for any schizophrenia‐related CpG sites. To look for an enrichment of CpG sites in modules of interest in schizophrenia GWAS regions, we used the manifest file provided by Illumina to identify all CpG sites within each of the 287 schizophrenia GWAS regions reported by Trubetskoy et al.^68^ Considering 395,274 CpG sites used in our network construction analysis as the background, we used a Fisher's exact test to investigate whether CpG sites in a module of interest were enriched in each of the 287 regions, performing this for (1) all CpG sites in a module and (2) hub CpG sites.

### 2.11 Data and code availability

We have archived the genome‐wide DNA methylation data and genotyping data for the 153 PITT‐ADRC samples on Synapse (syn23538600). All analytical scripts/code are available at https://github.com/UoE‐Dementia‐Genomics/AD‐Psychosis‐WGCNA.git

## 3 RESULTS

### 3.1 WGCNA identifies modules of co‐methylated loci associated with psychosis in AD

In the PITT‐ADRC cohort, we identified 99 modules ranging in size from 100 to 56,628 probes. Subsequently, we used a *t*‐test to compare the ME between AD+P and AD−P samples, identifying six nominally significant modules (darkgreen: *N* = 1023 probes, *p* = 0.046; firebrick4: *N* = 122 probes, *p* = 0.025; darkseagreen3: *N* = 105 probes, *p* = .012; magenta: *N* = 6368 probes, *p* = 0.030; grey60: *N* = 1830, *p* = 0.018; greenyellow: *N* = 5512 probes, *p* = 0.045). These six modules all showed a significant positive correlation between MM and PS (darkgreen: *r* = 0.12, *p* = 1.90 × 10^−4^; firebrick4: *r* = 0.21, *p* = 0.023; darkseagreen3: *r* = 0.36, *p* = 1.5 × 10^−4^; magenta: *r* = 0.14, *p* = 1.14 × 10^−28^; grey60: *r* = 0.21, *p* = 4.82 × 10^−20^; greenyellow: *r* = 0.10, *p* = 7.42 × 10^−14^) (Figures S3 to S8).

### 3.2 Darkgreen module was preserved and associated with AD ± P in BDR cohort

**FIGURE 2:**
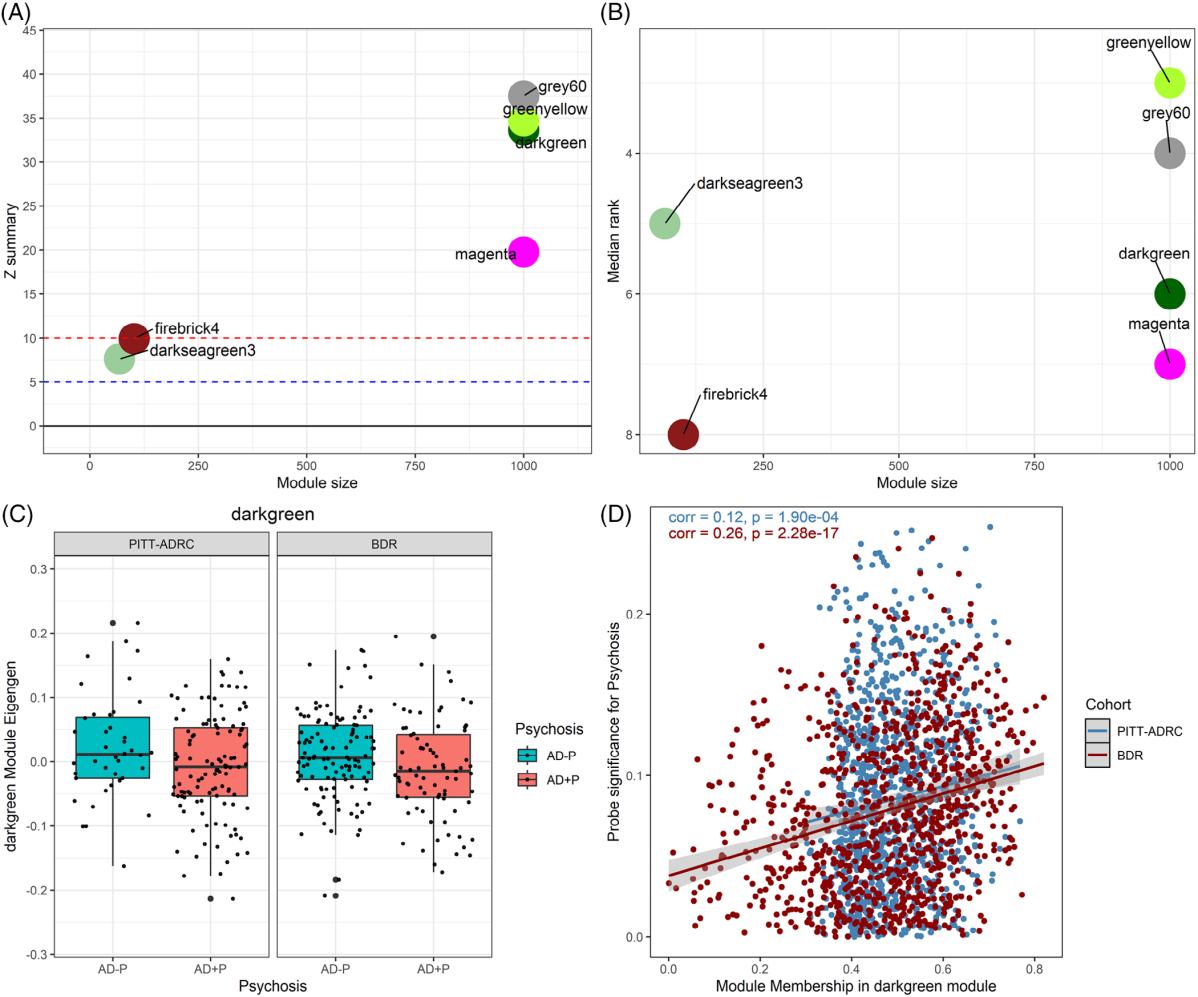
The darkgreen module was preserved in the validation cohort and associated with AD+P. (A) The Z‐summary for WGCNA module preservation analysis in the BDR validation cohort. (B) Median rank for WGCNA module preservation in BDR validation cohort. (C) Boxplot showing that ME for darkgreen module was significantly lower in AD+P compared to AD−P in both PITT‐ADRC (left plot) and BDR (right plot) cohorts. (D) Darkgreen module MM was correlated with PS in both PITT‐ADRC (blue) and BDR (red) cohorts. AD+P, Alzheimer's disease with psychosis; AD−P, Alzheimer's disease without psychosis; BDR, Brains for Dementia Research; ME, module eigengene; MM, module membership; PITT‐ADRC, University of Pittsburgh Alzheimer's Disease Research Center; PS, probe significance; WGCNA, weighted gene correlation network analysis.

To replicate our findings, we sought to examine whether these six modules associated with AD+P were preserved in an independent dataset. All six modules showed a moderate to high degree of preservation (Figure 2A‐B). However, when we compared the ME between AD+P and AD−P samples in the BDR validation cohort, only the darkgreen module showed a significant difference between AD+P and AD−P samples (*p* = 0.039, Figure 2C), also showing a significant correlation between PS and MM (*r* = 0.26, *p* = 2.28 × 10^−17^; Figure 2D).

### 3.3 Darkgreen module was enriched in excitatory and inhibitory neurons and in synaptic pathways

**FIGURE 3:**
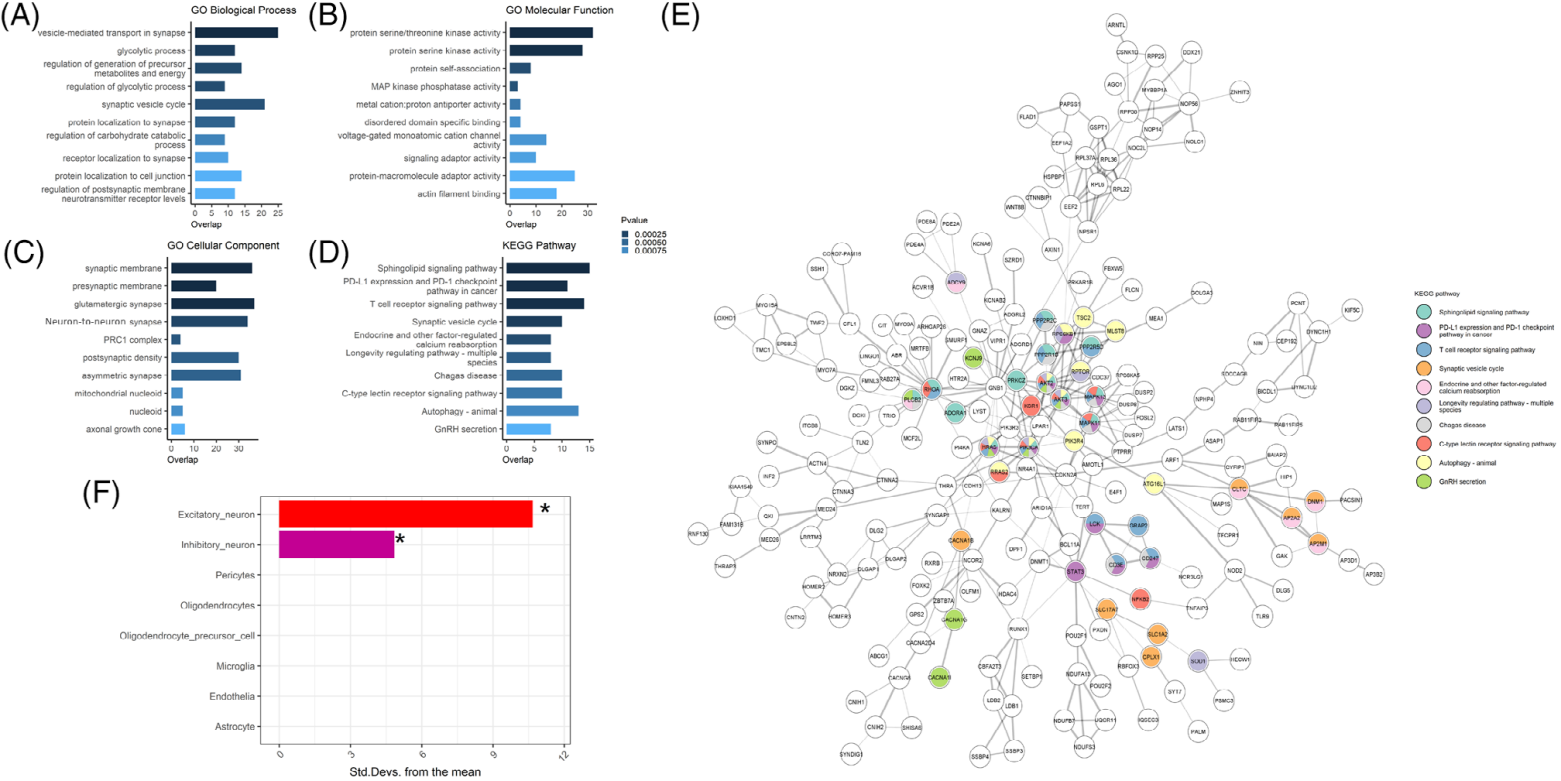
Enrichment analyses in darkgreen module for pathways and cell types. The 10 most significantly enriched GO biological process (A), molecular function (B), and cellular component (C) terms and KEGG pathways (D) in the darkgreen module. (E) PPI network for genes within 1 kb of each CpG in the darkgreen module. The PPI network was extracted from the string database with a confidence score >0.7. (F) Cell‐type enrichment analysis for CpGs in the darkgreen module using the ROSMAP single‐cell data, with the asterisk denoting a significant enrichment. KEGG, Kyoto Encyclopedia of Genes and Genomes; GO, Gene Ontology; PPI, protein–protein interaction; ROSMAP, Religious Orders Study and Memory and Aging Project.

Overall, for the 1023 CpG sites in the darkgreen module (Table S2), 784 (76.6%) had a RefSeq (Illumina) annotation, corresponding to 667 unique genes. None of these CpG sites overlapped with the previously reported Braak‐associated DMPs reported in a recent meta‐analysis of prefrontal cortex EWAS datasets,^33^ suggesting they are specific to AD+P rather than simply reflecting AD pathology. When we focus specifically on the 69 hub CpG sites in this module (ie, those with > 25% MM and PS), 59 had a RefSeq annotation, corresponding to 56 unique genes. GO analysis was performed on all annotated CpG sites in the darkgreen module, and we identified several nominally significant pathways enriched in the darkgreen module (Table S3, Figure 3A‐C), with the most significant GO term relating to the vesicle‐mediated transport in synapse (GO:0099003: *p* = 5.35 × 10^−5^). KEGG pathway analysis highlighted several nominally significant terms (Table S4, Figure 3D), the most significant being annotated to the sphingolipid signaling pathway (hsa04071: *p* = 6.02 × 10^−4^). The genes with physical interactions at the protein level in the PPI network were mostly related to T‐cell receptor signaling and Sphingolipid signaling pathways (Figure 3E), which were also the first and third most significant KEGG terms, respectively. It was not appropriate to perform pathway analyses on just the hub genes, given that there were only 56 unique genes in this list. Our cell‐type enrichment analysis using EWCE showed a significant enrichment for genes expressed by excitatory neurons and inhibitory neurons in all genes annotated to the darkgreen module (Figure 3F), whereas when we restricted this to just the hub genes, we observed a significant enrichment in excitatory neurons only.

### 3.4 mQTLs in darkgreen module co‐localize with SNPs associated with schizophrenia and educational attainment

**FIGURE 4:**
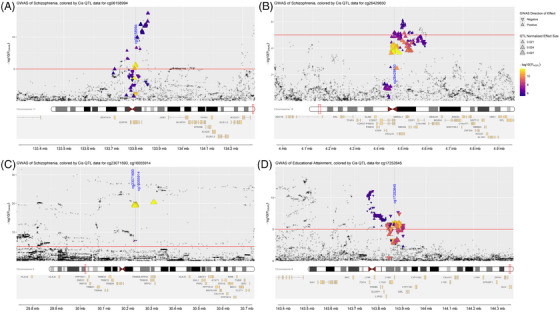
Co‐localization of *cis* mQTLs mapping to CpGs in darkgreen module with related traits. Overlapping SNPs between (A) a genomic locus associated with schizophrenia on chromosome 11 and *cis* mQTLs mapping to cg06158994, (B) a genomic locus associated with schizophrenia on chromosome 16 and *cis* mQTLs mapping to cg26429850, (C) a genomic locus associated with schizophrenia on chromosome 6 and *cis* mQTLs mapping to cg23071690 and cg16055914, and (D) a genomic locus on chromosome 8 linked to educational attainment and *cis* mQTLs mapping to cg17252645. The *x*‐axis shows genomic position, the *y*‐axis −log10(*P*) for GWAS. mQTL, methylation quantitative trait locus

Using the matched genotype and DNA methylation data for the 151 PITT‐ADRC that passed genotyping QC, we identified 7154 *cis* mQTLs (within ± 1 Mb of each CpG location; Table S5) corresponding to 135 unique CpG sites and 3449 *trans* mQTLs (Table S6) corresponding to 470 unique CpG sites within the darkgreen module, which were statistically significant (*p* < 1.0 × 10^−5^). We conducted a Bayesian test to examine co‐localization of these SNPs with genetic variants reported in GWAS for related traits (including AD, schizophrenia, panic disorder, anxiety, educational attainment, bipolar disorder, major depressive disorder, Lewy body dementia, AD+P, and reaction time) and then established a co‐localization *p* value for regions that co‐localized (eg, where H3 + H4 > 0.9). This highlighted four *cis* mQTLs co‐localized with genomic regions associated with schizophrenia (Table S7). This included 79 SNPs (lead SNP = rs4936215) co‐localized with cg06158994 (located within 1 kb of *IGSF9B*) on chromosome 11 (*p* = 0.001; Figure 4A), 143 SNPs (lead SNP = rs8048039) co‐localized with cg26429850 (located within 1kb of *CORO7‐PAM16* and *CORO67*) on chromosome 16 (*p* = 0.011; Figure 4B), and 43 and 11 SNPs (lead SNP = rs140365013) co‐localized with cg23071690 (a hub CpG site) and cg16055914, respectively (located within 1 kb of *HCG17* and *HLA‐L*) on chromosome 6 (*p* = 0.021, Figure 4C). We also identified one *cis* mQTL for cg17252645 (located within 1 kb of *LY6D* and *RPL11‐706C16.8*) on chromosome 8, which overlapped with a genomic region associated with educational attainment (lead SNP = rs2585183) (*p* = 0; Figure 4D). Our *trans* mQTL analysis also showed co‐localization of CpG sites in the darkgreen module with SNPs associated with schizophrenia and educational attainment (Table S7). We observed that cg02678159 (unannotated) on chromosome 3 co‐localized (*p* = 0.034) with 19 SNPs (lead SNP = rs17194490) and cg19711602 (annotated to *IQSEC3*) on chromosome 12 co‐localized (*p* = 0.04) with rs4766428, which are GWAS regions previously associated with schizophrenia. Similarly, cg18158739 (unannotated) on chromosome 14 co‐localized (*p* = 0.002) with six SNPs (lead SNP = rs73265641), associated with educational attainment.

### 3.5 CpGs in darkgreen module are enriched in schizophrenia GWAS regions

Given that we had demonstrated co‐localization of darkgreen module mQTLs with genetic variants associated with schizophrenia, we next explored whether there was an enrichment of darkgreen module CpG sites in schizophrenia EWAS and GWAS. We saw no shared CpG sites between the darkgreen module and the 2154 CpGs reported in the schizophrenia EWASs.^77^, ^78^ Next, we explored whether there was an enrichment of CpGs in the darkgreen module in each of the 287 GWAS regions recently reported for schizophrenia by Trubetskoy and colleagues.^68^ We saw a significant enrichment of darkgreen module CpG sites in 26 of these regions (Table S8), with four of these being Bonferroni significant. When we specifically focus on the 69 hub CpGs, we saw a significant enrichment of these in 10 of these regions, although none passed Bonferroni correction.

## 4 DISCUSSION

This study represents the most comprehensive study of brain DNA methylation in Alzheimer's disease psychosis. We identified six co‐methylation modules in the DLPFC associated with the presence of psychosis in a discovery cohort of > 150 well‐characterized *post mortem* AD brain samples, and we validated one of these modules (darkgreen) in an independent cohort of nearly 200 AD brain samples. This replicated module was enriched for pathways associated with synaptic functions, and cell‐type enrichment analysis showed these differentially methylated genes were enriched in excitatory and inhibitory neurons. Interestingly, a recent transcriptomic study on the PITT‐ADRC cohort highlighted a 9% decrease in the proportions of excitatory neurons in the DLPFC in AD+P compared to AD−P,^79^ with alterations in synaptic proteins previously reported in the PITT‐ADRC cohort.^49^ This is particularly interesting in the context of our study, as the most integral loci in the module (hub CpG sites) were significantly enriched in excitatory neurons. Alterations in the postsynaptic density proteome in AD+P were also recently described in the PITT‐ADRC cohort, with lower levels of proteins, particularly kinases, those regulating Rho GTPases, and those regulating the actin cytoskeleton.^80^ The current study provides evidence of synaptic dysfunction in AD+P via epigenetic (DNA methylation) mechanisms. Taken together, these studies highlight that synaptic dysfunction in AD+P is observed across multiple layers of genomic and cellular regulation. However, it is worth highlighting that GO and KEGG pathway analyses, as used in our study, are largely limited to canonical interactions, and this approach may not highlight alternative mechanisms that are not annotated.

We report that DNA methylation at a number of CpG sites in the darkgreen module was driven by mQTLs, with these co‐localizing with GWAS signals (both in *cis* and *trans*) reported for schizophrenia and educational attainment. This highlights potential regulatory relationships and genetic convergence at specific loci, implicating shared molecular mechanisms between AD+P and these traits. Interestingly, schizophrenia and educational attainment have been shown to have a strong genetic dependence with each other, with several genes having pleiotropic effects.^81^ In the context of the current study, shared genetic liability of AD+P with schizophrenia was previously shown, with a meta‐analysis of > 3000 AD cases reporting that AD+P individuals had a higher PRS for schizophrenia^41^ and with genetic overlap with a number of psychiatric disorders.^82^ Although a recent GWAS of AD+P utilizing 12,317 AD and 5445 AD+P subjects suggests that there are some genetic correlations for schizophrenia and bipolar disorder,^48^ the current study went beyond simple genetic correlations to investigate whether DNA methylation associated with psychosis in AD might be driven by genetic variation associated with relevant neuropsychiatric traits. Importantly, we identified a co‐localized GWAS signal for schizophrenia with genetically driven methylation patterns in AD+P in both *cis* and *trans*. We also demonstrated that our AD+P‐associated DNA signature was significantly enriched in four schizophrenia GWAS regions. This suggests transdiagnostic mechanisms for psychosis across the lifespan, extending previous findings linking schizophrenia and major depressive disorder PRS with AD+P,^26^, ^48^ by providing functional context. Interestingly, although we showed clear co‐localization between genetic risk variants for schizophrenia and genetically driven methylation signatures in AD+P, it is worth noting that some studies previously reported that the schizophrenia PRS was protective against AD+P.^25^ Although we were unable to explore this in the context of the current study given our sample size, one of the key strengths of our study is the integration of multiple levels of data, allowing us to perform co‐localization analyses. Looking forward, the examination of psychosis endophenotypes such as neuroimaging markers may provide more clarity and should be explored in the future.^83^

Over the years numerous epidemiological studies have explored the association between AD+P and educational attainment, although there have been inconsistencies between findings.^2^ However, previous genetic correlation studies showed an inverse relationship between education and AD+P risk,^48^ suggesting a genetic link between these two traits. Given that we have shown co‐localization of SNPs associated with educational attainment with methylomic alterations in AD+P, this may suggest a role for cognitive reserve in reducing psychosis risk in AD, which warrants further investigation.

It is worth noting that a limitation of our study is that it was performed on bulk brain tissue. In addition to reduced numbers of excitatory neurons, increased proportions of oligodendrocytes and endothelial cells have also been described in the DLPFC in AD+P in the PITT‐ADRC cohort.^79^ Although we controlled for the proportions of neuronal and non‐neuronal cells in our analyses, this did not allow for cell‐type‐specific investigations. Recent adaptations to fluorescence‐activated nuclei sorting (FANS) protocols now allow for the isolation of multiple brain cell types, including glutamatergic neurons, GABAergic neurons, oligodendrocytes, and microglia/astrocytes.^84^ The use of this method in combination with DNA methylomic profiling will be an important avenue for future studies to establish cell‐type‐specific DNA methylation signatures in AD+P. Our study leveraged the Illumina EPIC V1 array to identify co‐methylated loci associated with AD+P using WGCNA; however, although this array covers > 850,000 CpG sites, there is still limited coverage of some regions of the genome,^85^ so whole‐genome bisulfite sequencing would allow for the unbiased identification of co‐methylated loci in AD+P. In addition, the use of WGCNA to identify correlation networks is also not without limitations, for example, the resolution limit. This can be affected by the parameters selected during module generation and can lead to both overfitting and underfitting, resulting in the inappropriate clustering or the omission of loci within modules, respectively.^86^, ^87^ The approach we took was to use an independent validation cohort, taking forward for downstream analysis only the replicated module. However, this approach does mean we might not have followed up on some biologically relevant modules that were not replicated. In a similar vein, there are inherent differences between the discovery (PITT‐ADRC) and validation (BDR) cohorts, which may have been why we only replicated one PITT‐ADRC AD+P‐associated module in BDR. Although we strove to unify the two datasets by following the same data QC, normalization, and analysis pipelines, as well as harmonizing the criteria for rating psychosis, it is possible that demographic and cohort differences might have resulted in only one of the PITT‐ADRC AD+P‐associated modules being replicated. When we generated modules in the BDR cohort, we saw that none of the AD+P‐associated modules replicated in the PITT‐ADRC cohort. This might have been due to the lower number of *ante mortem* neuropsychiatric assessments available in BDR compared to PITT‐ADRC and was one of the reasons why we chose the PITT‐ADRC cohort for discovery. Nonetheless, as we were able to replicate one of the PITT‐ADRC modules (darkgreen) in the BDR cohort, this represents a robust methylation signature of AD+P. It will be of interest to further explore this module in additional well‐characterized cohorts as these become available in the future. Our study has used Caucasian samples, and future studies should also explore whether our findings are generalizable to other ethnic backgrounds. This is particularly important as it has been reported that the prevalence of psychotic symptoms is significantly higher in African Americans than Caucasians in the moderate to severe stages of AD.^88^

A number of different epigenetic mechanisms regulate gene transcription, including a variety of histone modifications and non‐coding RNAs. Looking to the future, it will be important to explore these different processes, ideally in isolated cell types. We identified important pleiotropic relationships in our study that certainly warrant further study; however, an inherent limitation of epigenetic studies in *post mortem* tissue is that it is not possible to reveal causal mechanisms. However, our study does suggest that DNA methylomic alterations in AD+P are enriched in synaptic genes and supports previous studies that reported alterations in excitatory neurons in AD+P. As such, this could be a novel treatment avenue for AD+P, particularly given that a recent trial in AD focused on modulating synaptic vesicle glycoprotein 2A (SV2A).^89^ Although we found that *SV2A* did not reside within 5 kb of the CpG sites comprising the darkgreen module, our study does reinforce that methylomic alterations in synaptic genes are observed in AD+P, so this treatment could still be explored for its therapeutic efficacy in psychosis in AD. Our study used unique sample cohorts that underwent neuropsychiatric investigations during life to enable the largest study of DNA methylation in AD+P brains to date, integrating genetic and epigenetic data.

## CONFLICT OF INTEREST STATEMENT

Clive Ballard has received grant funding and honoraria from Acadia Pharmaceuticals, honoraria from Bristol Myers Squibb, Exciva, and AbbVie related to AD+P. The other authors declare that they have no conflicts of interest. Author disclosures are available in the supporting information.

## CONSENT STATEMENT

This study used *post mortem* brain tissue collected at designated brain banks. Consent for autopsy for research purposes for the PITT‐ADRC Brain Bank was provided at the time of death by the next of kin in accordance with a protocol approved by the Committee for Oversight of Research and Clinical Training Involving Decedents. BDR participants in this study had detailed *ante mortem* clinical assessments and as part of this provided informed consent.

## Supporting information



Supporting Information

Supporting Information

Supporting Information
