# Supplementary material for: A brain DNA co‐methylation network analysis of psychosis in Alzheimer's disease
Source: Alzheimers Dement. 2025 Feb 12;21(2):e14501. doi: 10.1002/alz.14501 (PMC11815327; doi:10.1002/alz.14501)

Supplementary Figure 1: Hierarchical Clustering Analysis of PITT-ADRC Cohort Samples. The effects of confounding variables (age, sex, Braak NFT stage, estimated cell type proportion and plate) were regressed out of the DNA methylation data prior to hierarchical clustering analysis using (**A**) Euclidean distance and (**B**) Correlation distance to identify potential outliers, with no consistent samples identified between the methods.

A

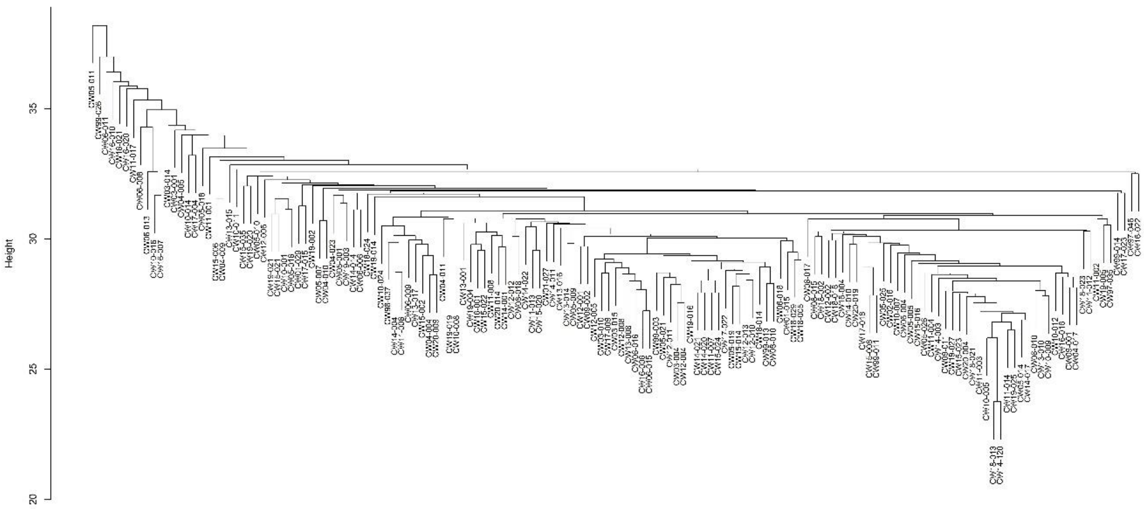

B

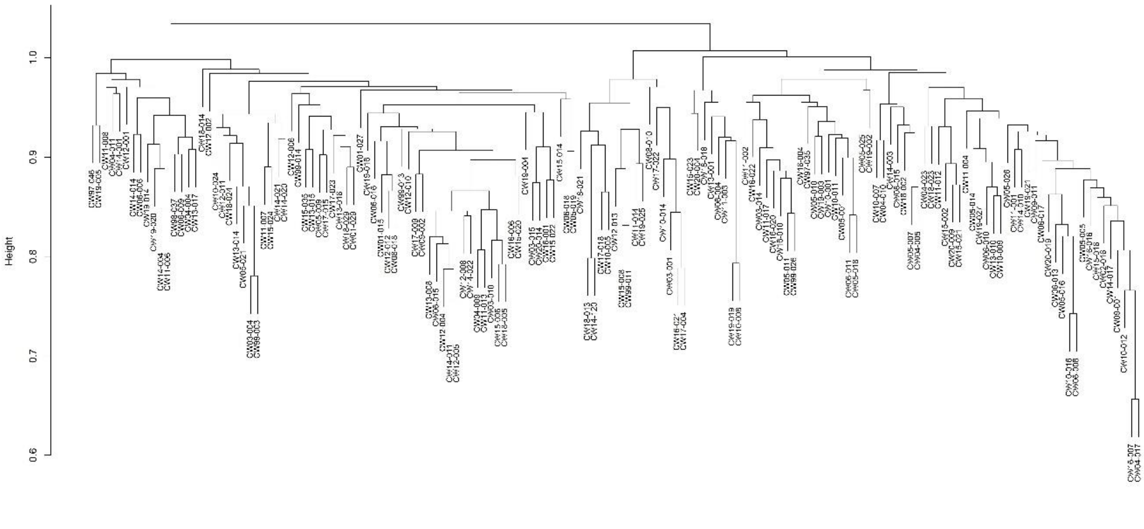

Supplementary Figure 2: Selection of Soft Threshold. (**A**) Scale free topology fitting index and (**B**) Mean of connectivity for multiple soft thresholding powers in PITT-ADRC data. The soft threshold value was set to three, which is achieved with a signed R^2 of 0.882 and a mean connectivity of 982.

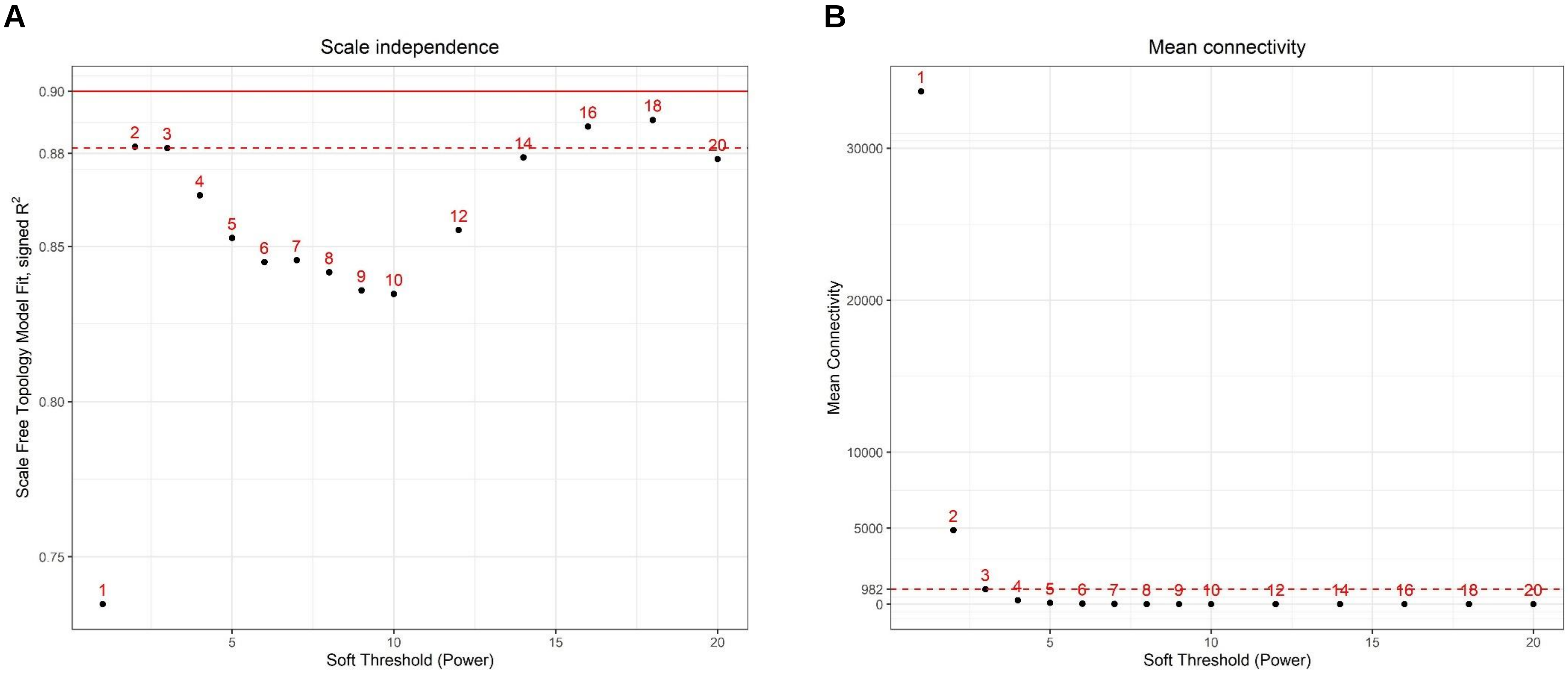

Supplementary Figure 3: The darkgreen module was significantly associated with the presence of psychosis in AD in the PITT-ADRC cohort. (**A**) There was a significant difference in the module eigengene value between the AD+P and AD-P groups (P = 0.046). (**B**) There was a significant correlation between the probe significance and module membership (r = 0.12, P = 1.90 x 10^{-4}).

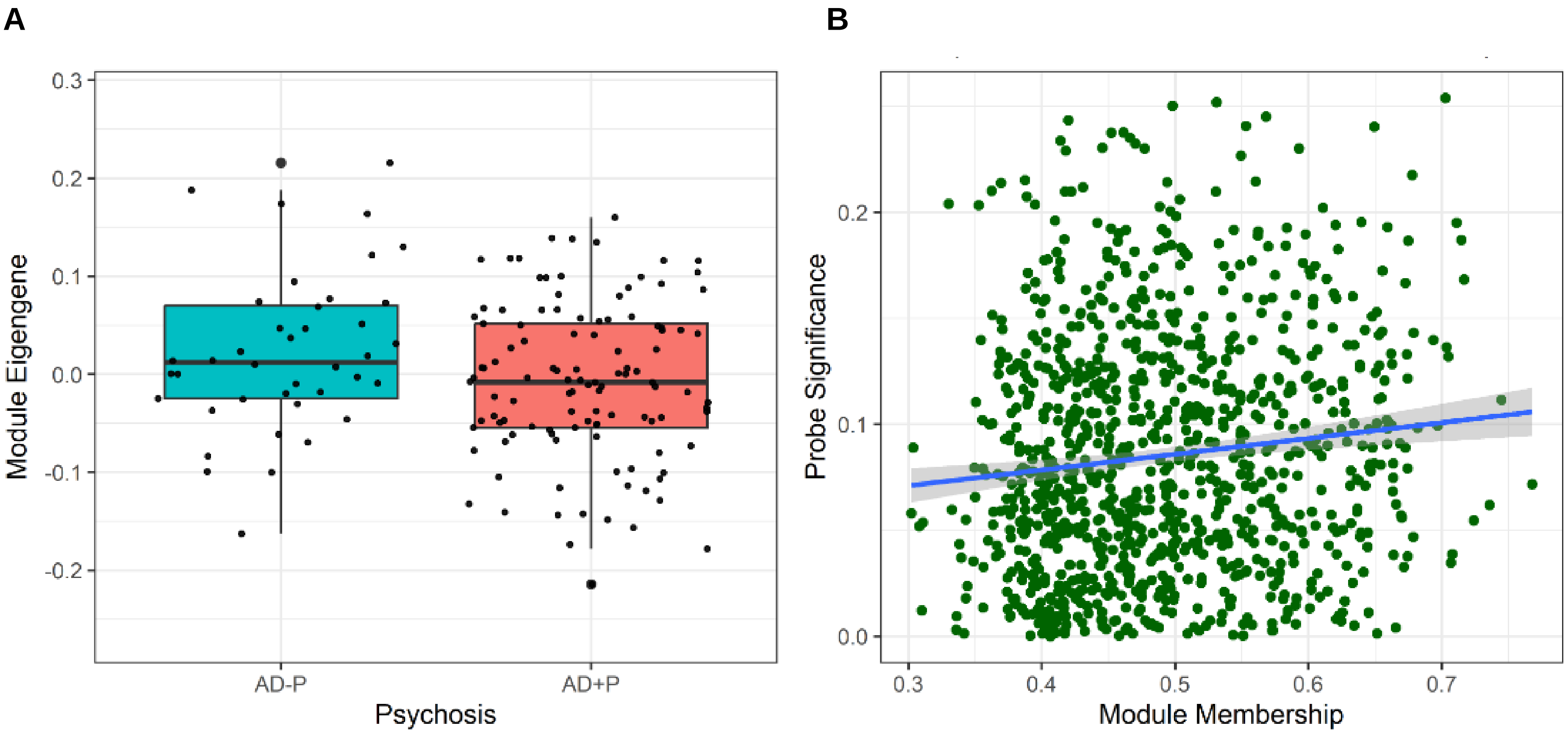

Supplementary Figure 4: The firebrick4 module was significantly associated with the presence of psychosis in AD in the PITT-ADRC cohort. (**A**) There was a significant difference in the module eigengene value between the AD+P and AD-P groups (P = 0.025). (**B**) There was a significant correlation between the probe significance and module membership (r = 0.21, P = 0.023).

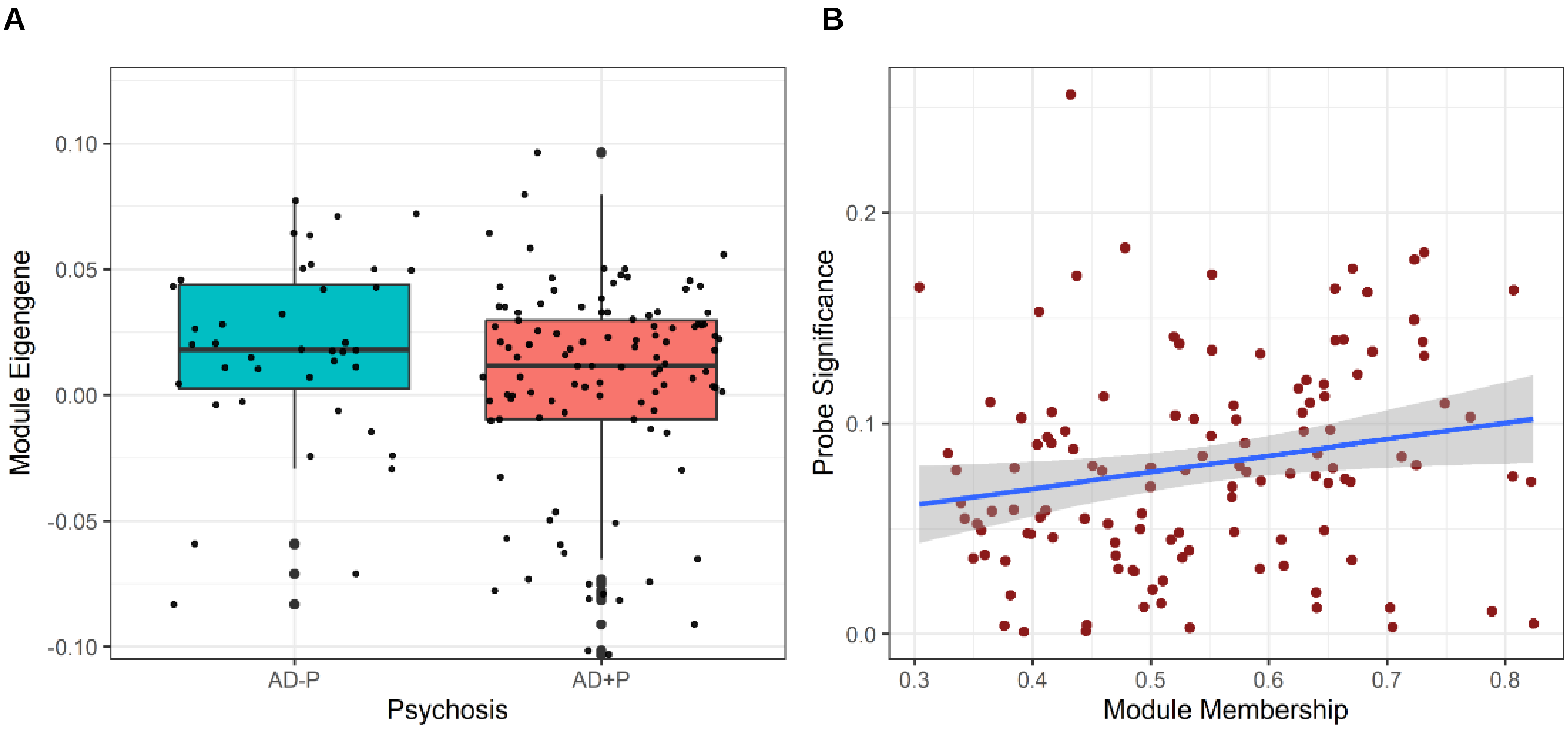

Supplementary Figure 5: The darkseagreen3 module was significantly associated with the presence of psychosis in AD in the PITT-ADRC cohort. (**A**) There was a significant difference in the module eigengene value between the AD+P and AD-P groups (P = 0.012). (**B**) There was a significant correlation between the probe significance and module membership (r = 0.36, P = 1.54 x 10^{-4}).

A **B**

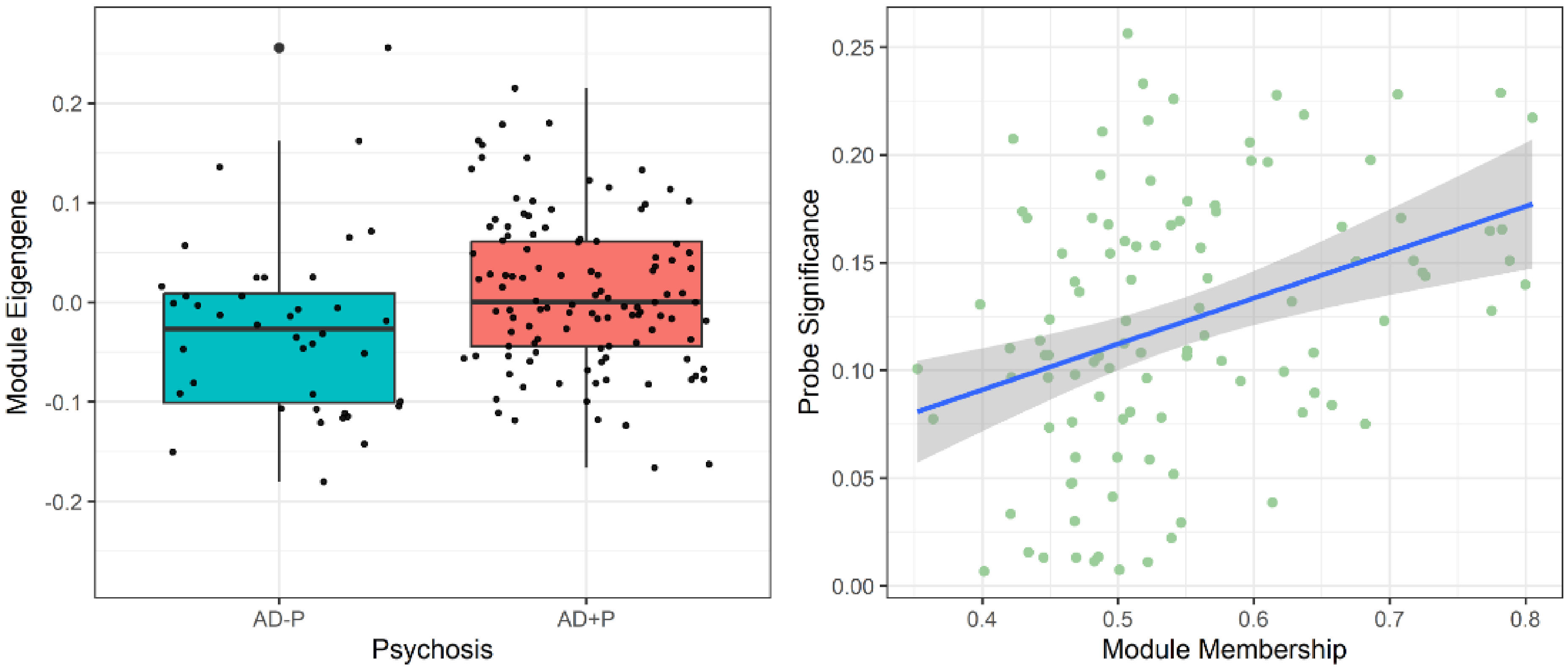

Supplementary Figure 6: The magenta module was significantly associated with the presence of psychosis in AD in the PITT-ADRC cohort. (**A**) There was a significant difference in the module eigengene value between the AD+P and AD-P groups (P = 0.030). (**B**) There was a significant correlation between the probe significance and module membership (r = 0.14, P = 1.14 x 10^{-28}).

A **B**

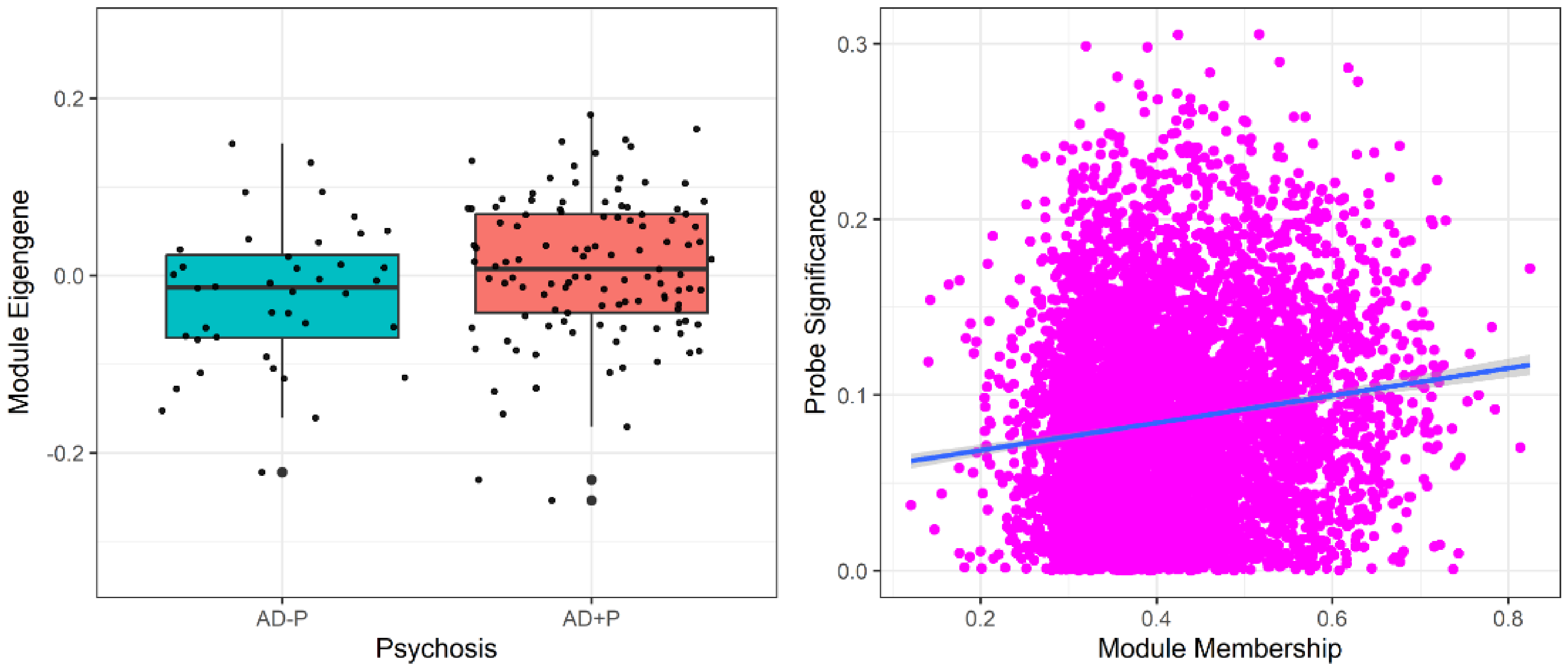

Supplementary Figure 7: The grey60 module was significantly associated with the presence of psychosis in AD in the PITT-ADRC cohort. (**A**) There was a significant difference in the module eigengene value between the AD+P and AD-P groups (P = 0.018). (**B**) There was a significant correlation between the probe significance and module membership (r = 0.21, P = 4.82 x 10^{-20}).

A **B**

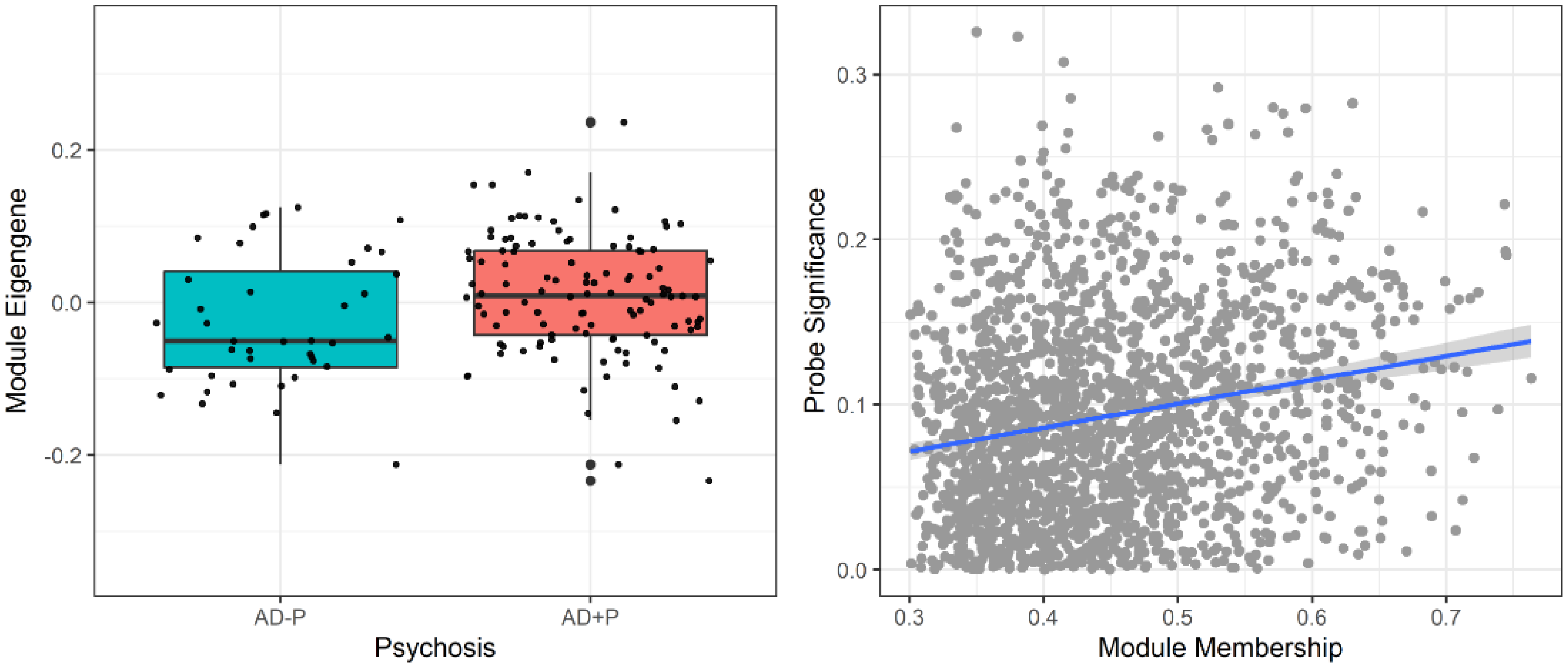

Supplementary Figure 8: The greenyellow module was significantly associated with the presence of psychosis in AD in the PITT-ADRC cohort. (**A**) There was a significant difference in the module eigengene value between the AD+P and AD-P groups (P = 0.045). (**B**) There was a significant correlation between the probe significance and module membership (r = 0.10, P = 7.42 x 10^{-14}).

A

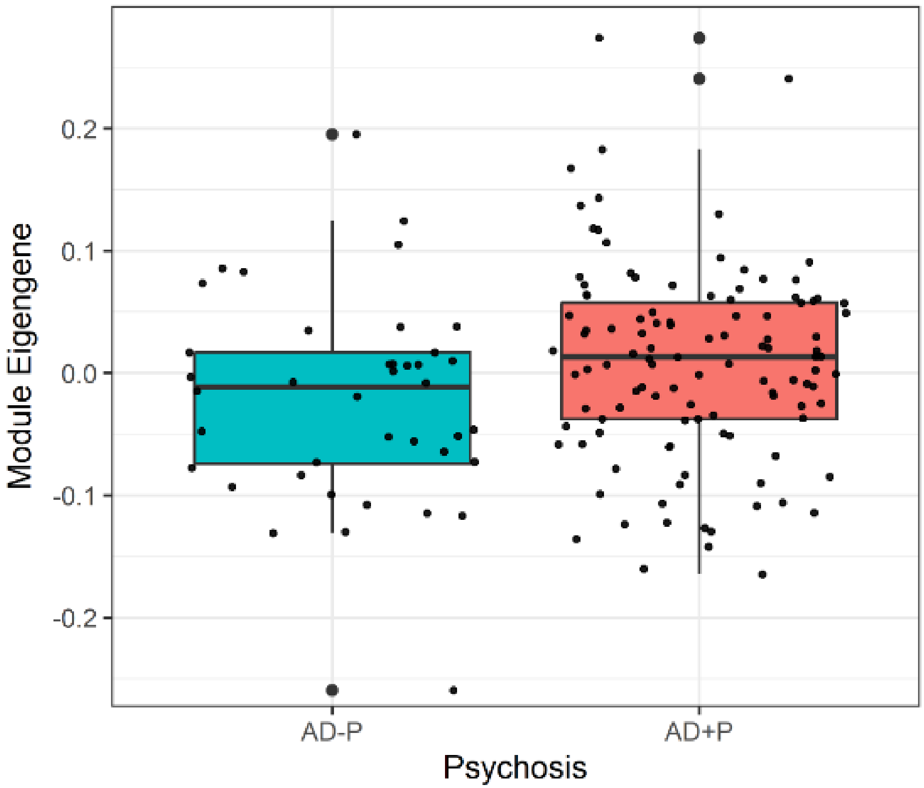

B

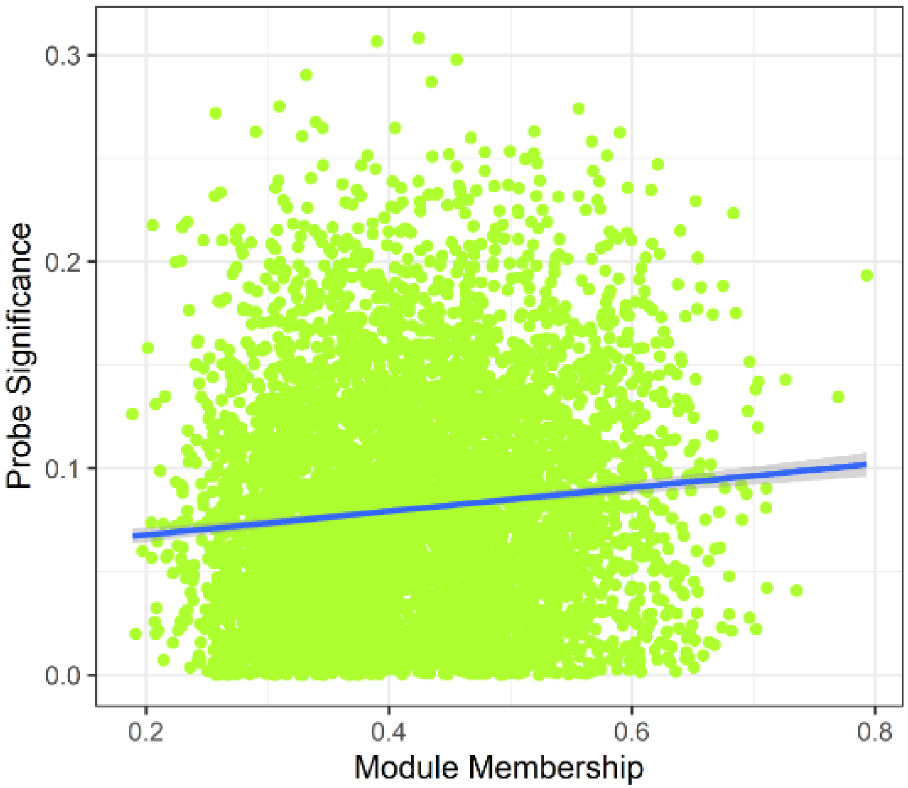

Supplement: Supplementary file 1 — Supporting Information [file ALZ-21-e14501-s003.pdf]
